# BacDive—the Bacterial Diversity Metadatabase

**DOI:** 10.1093/nar/gkt1058

**Published:** 2013-11-07

**Authors:** Carola Söhngen, Boyke Bunk, Adam Podstawka, Dorothea Gleim, Jörg Overmann

**Affiliations:** Leibniz Institute DSMZ—German Collection of Microorganisms and Cell Cultures, Inhoffenstr. 7B, 38124 Braunschweig, Germany

## Abstract

BacDive—the Bacterial Diversity Metadatabase (http://bacdive.dsmz.de) merges detailed strain-linked information on the different aspects of bacterial and archaeal biodiversity. Currently (release 9/2013), BacDive contains entries for 23 458 strains and provides information on their taxonomy, morphology, physiology, sampling and concomitant environmental conditions as well as molecular biology. Where available, links to access the respective biological resources are given. The majority of the BacDive data is manually annotated and curated. The BacDive portal offers an easy-to-use *simple search* and in addition powerful *advanced search* functionalities allowing to combine more than 30 search fields for text and numerical data. The user can compile individual sets of strains to a *download selection* that can easily be imported into nearly all spreadsheet applications.

## INTRODUCTION

BacDive—the Bacterial Diversity Metadatabase (http://bacdive.dsmz.de) was initiated by the Leibniz Institute DSMZ—German Collection of Microorganisms and Cell Cultures (DSMZ, http://www.dsmz.de) in April 2012 and is maintained and curated at the DSMZ ever since. BacDive covers a broad range of detailed information on a wide variety of bacterial and archaeal strains describing their individual properties and characteristics. Areas covered by BacDive comprise the taxonomy, morphology, physiology, cultivation, geographic origin, application, interaction or sequences for genomes and 16S rRNA deposited at the International Nucleotide Sequence Database Collaboration (INSDC) (1). These strain-associated metadata are all connected to strains and their appropriate sources of supply. The information in BacDive is predominantly annotated manually. The source material for the annotation also includes detailed internal descriptions of culture collections, which are not publicly available. Likewise, expert-compiled compendia on strains are extracted and annotated. Furthermore, information is extracted from the relevant primary scientific literature. Although predominantly manually annotated, a small amount of data is recovered by automatic text processing. In this latter case, each data point derived by text mining methods is clearly marked and can be displayed or hidden from the detailed view of the dataset for each strain and can be excluded during export of data. As an additional functionality, individual data annotation is currently tested at an initial alpha stage for BacDive. The *simple search* option of the portal allows queries for all strains belonging to a species by entering full-length or parts of the species name. The *advanced search* function is employed to conduct sophisticated queries by combining more than 30 search fields for text and numerical data fields. The *advanced search* allows also an exclusion search in user-selected fields. BacDive is therefore an ideal central entry point for a quick retrieval of detailed information on distinct strains and at the same time suitable to perform large-scale queries for comparative and simultaneous analyses of a multitude of strains.

As one of the world’s major culture collections, the Leibniz Institute DSMZ is a suitable host for BacDive. DSMZ is an active member of global bioresource centre networks such as the World Federation for Culture Collections (WFCC, http://www.wfcc.info) and publishes information on its biological resources for years via the Global Biodiversity Information Facility (GBIF, http://www.gbif.org), the DNA Bank Network (http://www.dnabank-network.org) or via contributions to the strain-associated catalogue of the StrainInfo bioportal (www.straininfo.net). The launch of the BacDive portal represents a new and central element of the portfolio of the biological resource centre Leibniz Institute DSMZ since it yields access to information about cultured microbial biodiversity, which is currently not provided anywhere else. StrainInfo offers comprehensive aggregated information on deposited strains and their exchange history. For this purpose, the catalogue information of many culture collections worldwide are integrated. The corresponding culture collection catalogue pages can be displayed for StrainInfo entries within the webportal. In addition, StrainInfo offers links to literature connected to the strain. Information about physiological or morphological characteristics is not part of StrainInfo. BacDive aims to provide detailed information on each strain including its physiological and morphological features, annotated as structured database content. This gives the user a quick overview on the strains characteristics and enables the user to filter the information for all strains according to particular attributes.

Within the German GBIF network GBIF-D the Leibniz Institute DSMZ constitutes the node for Bacteria and Archaea (http://www.gbif.de/prokaryotes). The main objective of the network is to mobilize organism-linked information in order to make them publicly available. For this purpose, the mobilized data are continuously mapped to the ABCD (http://www.bgbm.org/TDWG/CODATA/schema/default.htm) data standard and published via the GBIF Data Portal (http://data.gbif.org). The GBIF Data Portal offers observational data for all species within the tree of life. In contrast to GBIF, the novel BacDive database provides a multitude of different metadata while focusing on microorganisms. Consequently, the means of presentation and retrieval of data is adjusted to the needs of research related to the field of microbiology. The relational database behind BacDive was constructed by defining over 400 potential data fields for handling data that cover all aspects of microbial diversity. At the same time, the very different types of data to be incorporated in the database rendered it impossible to adopt a single data standard throughout. So far, several data standards focusing on structuring and publishing taxon- or sample-associated data such as ABCD, DarwinCore (http://rs.tdwg.org/dwc) or MIxS (2) exist. However, essential features for the description of bacteria (phenotypes, cultivation, isolation) are missing from or cannot be mapped completely to the individual standards. In an attempt to keep as consistent as possible with existing standards, we adapted and integrated the appropriate data fields from existing standards into the BacDive set of data fields. The largest overlap of BacDive data fields exists with the MIxS and ABCD schemes. For these fields the original field syntax and data structure were used also in BacDive. Some progress has recently been made by the respective working groups of GBIF and the Genomic Standards Consortium (GSC, http://gensc.org) to harmonize different data standards within the fields of biodiversity, microbiology and genomics (3).

## BACDIVE DATA

### BacDive content

The BacDive platform system provides a wide range of metadata for each bacterial and archaeal strain. The current (9/2013) BacDive release covers more than 23 400 strains that are distributed over more than 1800 genera and 9000 species. Nearly 6000 of the strains in BacDive represent the type strains of their species. BacDive strives to provide information on all aspects of microbial diversity and therefore encompasses over 400 predefined data fields. To date, 179 of the data fields are in use within the BacDive relational database. All BacDive data fields are assigned to one of seven thematic sections (Table 1).

**Table 1.** The seven sections of BacDive and the number of entries per section

		Entries per section^a^	Entries per section^a^	increase
Section name	Selected subjects of the section	Release 9/2012	Release 9/2013	2012–13 (%)
Name and taxonomic classification	Domain, phylum, class, family, genus, species and subspecies (if the referring taxonomic ranks are already assigned), available the full scientific name and type strain status	18 157	23 458	29.2
Morphology and physiology	Utilized substrates, known produced compounds, tolerance level towards for several substances/antibiotics, murine types, lysis/decomposition ability	4053	11 521	184.3
Culture and growth conditions	Cultivation media compositions, growth temperatures, pH, salt concentrations, fumigation, lightning constrains	20 634	21 605	4.7
Isolation, sampling and environmental information	Geographic location (continent, country, city and further details, e.g. sea or region, geographic coordinates), environmental conditions at sampling time, utilized enrichment media	15 449	20 769	34.4
Application and interaction	Medical, biotechnical or industrial application, strain-associated patents, risk group classification, biotic relationship, potential host relations	0	14 639	100.0
Molecular biology	Genotype information (connected to sequencing results, secondary and tertiary sequence analysis), e.g. INSDC sequence accession numbers, sequence length, GC-content, applied analysis methods	13 609	14 591	7.2
Strain availability	Depository history, holding biological resource centre, culture collection identifiers	17 856	18 459	3.4
∑ overall entries		89 758	125 042	39.3

^a^Distinct combination of strain, reference, data entry.

In order to provide high quality content throughout, BacDive data are predominantly annotated manually and each entry is provided together with its source reference. Currently, the majority of references are derived from comprehensive strain descriptions by researchers or by biological resource centres holding the respective strains. However, the source material for BacDive also includes an increasing fraction of content derived from basic microbiological research involving the strains. For this purpose, annotation of information present in the primary scientific literature has been started. Additionally, we have implemented additional functionalities for individual data annotation in BacDive. This extension is currently tested in house. Since the integration of new data is a central objective of BacDive and is a continuous process, potential data providers are contacted by the BacDive team. Similarly, proposals for the integration of relevant collection descriptions are highly encouraged. BacDive offers the opportunity to publish datasets in an highly integrated database as well as in the connected resources such as the GBIF data portal, which guarantees a high visibility.

All available information for each strain is structured and displayed according to sections. The scope of each section is described in the following.

### Name and taxonomic classification

The taxonomic classification of strains in BacDive follows *Prokaryotic Nomenclature up-to-date* (http://www.dsmz.de/bacterial-diversity/prokaryotic-nomenclature-up-to-date.html) that is maintained and curated in house. This database comprises a compilation of all names of bacteria and archaea, which have been validly published according to the Bacteriological Code (4) since 1 January 1980. Also included are all subsequent changes in nomenclature, which have been validly published (5). *Prokaryotic Nomenclature up-to-date* is expert curated and published at the Leibniz Institute DSMZ and routinely updated following the release of each new issue of the *International Journal of Systematic and Evolutionary Microbiology* (*IJSEM*). Based on this information, BacDive offers curated information on the taxonomic ranks domain, phylum, class, order, family, genus, species and subspecies name that are already assigned to the strain. When available the full scientific name and type strain status are listed.

### Morphology and physiology

The section *morphology and physiology* encompasses the majority of active fields. It is covered by 108 out of the currently 179 BacDive data fields in the database. Within this section information about growth substrates, the compounds produced, the tolerance levels for inhibitors and antibiotics, the peptidoglycan types (1202 entries), the strains ability of yeast lysis (729 entries), and cellulose (17 entries) or chitin (319 entries) degradation can be found.

Since several physiological characteristics are meanwhile screened via laboratory test kits, BacDive also offers annotated test kit results. Data generated through the commercial systems bioMérieux API® ZYM (6) (344 entries), API® Coryne (219 entries) and API® 20E kit (298 entries) are currently included. These datasets will be extended by data from additional, commonly used test kits where feasible. Besides listing the results of tested activities of enzymes this information is mapped to valid EC numbers and entries are linked to the BRENDA (7) enzyme information system. The morphological characteristics of the strain that are provided by BacDive comprise the cell and colony morphology on different cultivation media and microphotographs. Currently, the BacDive portal contains more than 1500 images of 570 strains. For strains forming multicellular associations (6388 entries) the available description or photographic documentation is provided.

### Culture and growth conditions

In the section *culture and growth conditions* BacDive focuses on information on the culture conditions, such as media composition, optimum growth temperature, pH and salt concentration. This subset currently contains 21 605 entries. Although related to the potential physiological capabilities (see previous paragraph) this section gives easy access to data on suitable cultivation conditions. In order to further structure the media ingredients, we aim to link the media recipes to chemical ontologies, e.g. ChEBI (8) in future releases.

### Isolation, sampling and environmental information

The location and date of sampling and isolation as well as the concomitant environmental conditions are recorded in fields assigned to the section *isolation, sampling and environmental information*. The documentation of the geographic location may include the name of continent, country, city and further details, e.g. a particular ocean or region. BacDive currently comprises 17 856 entries with geographic information. This section also includes data fields about the sample type (20 163 entries) and the given environmental conditions at the time of sampling. Available information on geographic latitude and longitude is automatically linked to OpenStreetMap (http://www.openstreetmap.org). In this section the enrichment media and conditions utilized are recorded as well.

### Application and interaction

The section *application and interaction* comprises data fields on medical, biotechnical or industrial applications of the strain and associated patents. Furthermore, the classification of the strains according to biological risk groups is provided (14 639 entries) together with the underlying legislation and the relevant legal bodies. The section also has predefined fields for observed biotic relationship or information on potential interactions with eukaryotic or prokaryotic hosts.

### Molecular biology

The section *molecular biology* includes data fields describing the genotype and information connected to whole genome or 16S rRNA sequencing results, secondary and tertiary sequence analysis as well as the predicted phenotypic capabilities and the potential regulation of the strains. Currently, BacDive holds more than 8000 strain-associated INSDC sequence accession numbers for genomes and 16S rRNA, partially accompanied by the information on sequence length. Additionally, BacDive offers more than 6500 entries regarding GC-content. For more than 2600 entries the applied analysis methods are stated. In future releases of BacDive, the section *molecular biology* will be specifically augmented by additional active data fields and functionalities in order to further interlink physiological with genomic information.

### Strain availability

The section *strain availability* includes fields with information about the actual source and the history of deposit for each strain. Available links are offered to other microbial resource centres that distribute the prokaryotic strain.

### Myxobacteria collection description

The comprehensive description of a collection of Myxobacteria can serve as a prominent example for the annotation work that is typically conducted in the BacDive project. This integral part of BacDive was established in 2012 and 2013 and is based on the world’s largest collection of myxobacterial strains assembled by Prof. Dr Hans Reichenbach at the former Gesellschaft für Biotechnologische Forschung (GBF) in Braunschweig, now Helmholtz Centre for Infection Research (HZI). The collection comprises more than 6000 documented strains, of which more than 2800 are included in the DSMZ microorganism strain catalogue. Myxobacteria are common in soils and animal dung and have an extraordinary life cycle. The cells swarm by gliding over the substrate surface and form fruiting bodies via cell aggregation under conditions of starvation (9). The genome of myxobacteria can exceed a size of 13 Mbp and typically harbours numerous genes for complex and novel biosynthetic pathways for the synthesis of secondary metabolites.

Besides the collection of myxobacteria itself, a large archive of index cards consisting of more than 12 500 pages had been deposited at the Leibniz Institute DSMZ by H. Reichenbach. The index cards contain descriptions of the growth of pure cultures, the morphology of fruiting bodies, details of the geographic origin and data regarding the habitat of the strains. Furthermore, the documentation comprises photographic images of these strains showing the morphology of their fruiting bodies. Based on a mutual agreement with the HZI, all this unique information for the first time could be digitized, structured, combined with taxon-associated biodiversity data and made publicly accessible. In an initial step, the whole documentation was been digitized and the digital representation was preannotated by means of automated text analysis. The subsequent manual annotation and integration into the database is still ongoing. The current release (9/2013) of BacDive already contains information on more than 4700 strains. This includes also 1197 photographic images that document the distinct and rich morphology of fruiting bodies of these bacteria as well as the morphological diversity of gliding and swarm formation.

## THE BACDIVE PORTAL

**Figure 1.**
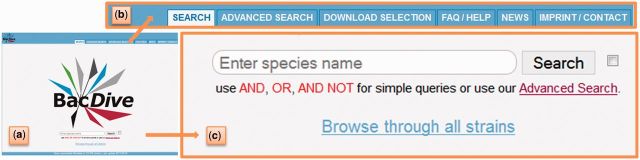
*Simple search*. BacDive portal entrance (**a**) with a clear user interface provides quick access through its navigation bar on the top of the site (**b**) to *simple search (SEARCH)*, *ADVANCED SEARCH* and *DOWNLOAD SELECTION*. Also at the navigation bar the FAQ/HELP, NEWS and IMPRINT/CONTACT can be called anytime. A free text search is activated by selecting the check box right to the *simple search* query field (**c**).

The BacDive portal offers a quick and unified access to strain information using either a *simple search* or an *advanced search* through its navigation bar (Figure 1). Search results within BacDive refer to *strain detail views* for each of the listed strain. More complex query functionalities and the export of user compiled *download selections* are available for both search routines in BacDive.

### Basic search functionalities

A *simple search* within BacDive can be performed starting right at the portal entrance site (Figure 1c). It allows for queries by entering part of the entire species name. Already while entering the first letters, matching species names are suggested in a drop down menu. This menu also displays the number of matching strains. In those cases where a submitted query for a species name leads to an empty result set, the system suggests names that are similar to the entered search term. The suggested species names are pooled together according to the lowest value of the Levenshtein distance (10) compared to the entered phrase. By selecting the check box right to the query field (Figure 1c) the free text search within several main fields of the BacDive database is activated. These fields include content in text format from all thematic sections of BacDive. The *simple search* function also allows for combinatorial queries on the species name using ‘AND’, ‘OR’ and ‘AND NOT’. The same query functionality offered by the BacDive portal entrance site can also be found in the upper left side of each *strain detail view* (Figure 3, top left).

### Advanced search

**Figure 2.**
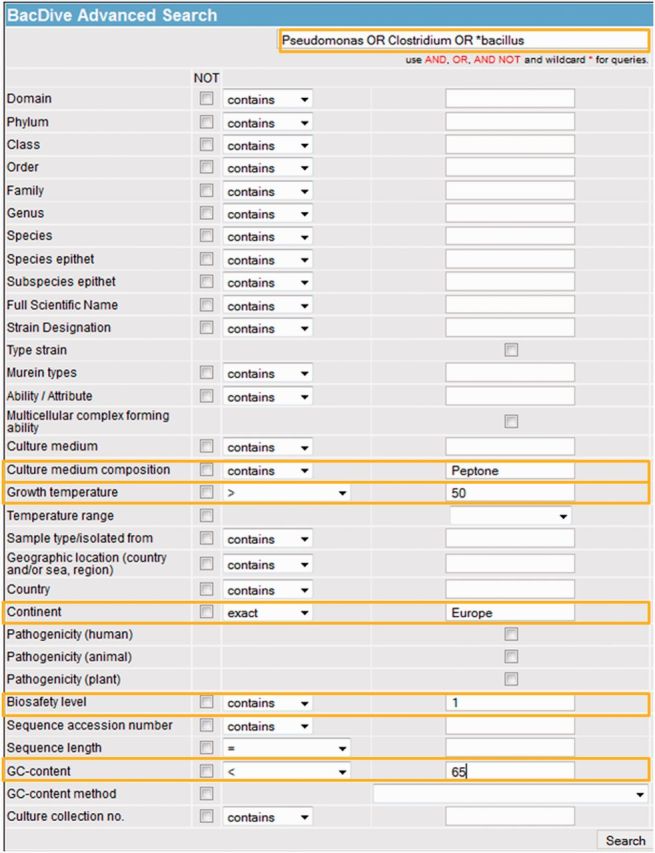
*Advanced search*. All search fields feature short tool tips containing compact explanations of the scope of the search fields, which are displayed on mouse-over. The query pattern (search terms highlighted in yellow) shown retrieves all strains with their species name containing ‘Pseudomonas OR Clostridium OR *bacillus’, having assigned the biosafety level 1, growing at a temperature higher than 50°C, on media containing ‘Peptone’, GC-content <65 mol% and being sampled in Europe. This query retrieves 15 strains.

The *advanced search* function offers large-scale queries combining several data fields for comparative analyses of a multitude of strains. The user can individually compile sophisticated queries by combining more than 30 search fields for text and numerical data fields (Figure 2). Each field for which valid phrases or selections are entered is included in the submitted query. Text-based data fields may be queried using individual phrases combined with the provided ‘begins with’, ‘ends with’, ‘contains’ and ‘exact’ filters. Numerical data fields can be filtered by ‘=’, ‘<’, ‘>’ or by a range when choosing the option ‘between min–max’. Data fields containing ‘true’ or ‘false’ just have to be selected by one click. Furthermore, the user may explicitly exclude certain phrases or values from text and numerical data fields. The very first field on the top of the *advanced search* form allows for combinatorial queries using ‘AND’, ‘OR’ and ‘AND NOT’ as described above for the *simple search* function. Additionally, search phrases can be combined with ‘*’ as wildcard for one or more characters.

**Figure 3.**
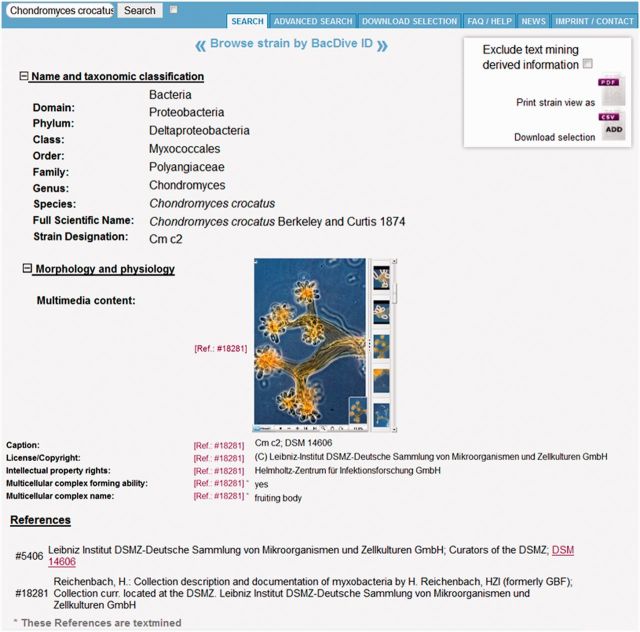
*Strain detail view*. The sections ‘Culture and Growth conditions’, ‘Isolation, sampling and environmental information’ as well as ‘Application and Interaction’ are hidden in this view for better clarity. A grey ‘*’ symbol besides the reference identification number indicates content derived from text mining.

### Strain detail view

**Figure 4.**
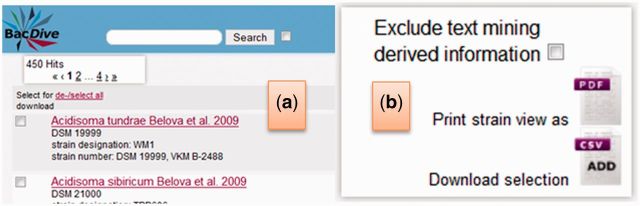
Results page and export panel. After submitting a query, a result set is generated and displayed as a list (**a**). Strains can be selected using checkboxes besides the list (a) or via the *export panel* (**b**) integrated in the *strain detail view*.

For each strain listed on the search results page (Figure 4a) a *strain detail view* can be displayed (Figure 3). It contains all available information stored in the database and is structured according to the sections listed in Table 1. Every section can be collapsed or unfolded by clicking on the ‘–’ or ‘+’ symbol, respectively, before its name. Each field and section name is explained by short tool tips, which are displayed by mouse-over. The tool tips are containing compact explanations of the scope of the fields and sections. On the bottom of the *strain detail view* the source references of the annotation are listed. The *strain detail view* may also comprise image material, especially for the new content from myxobacteria (see above) and important type or reference material. Functions to display multiple images in a single viewer along with advanced zooming features are offered. Most of the images are accompanied by comprehensive captions.

An export panel is integrated within the *strain detail view* (Figure 3, top right and Figure 4b in detail). This panel offers options to select certain strains for CSV export or PDF printing. Similar to the *strain detail view* itself, text mining derived information also can be excluded in the export panel. Text mining derived content is always indicated by a grey ‘*’ symbol besides the reference number.

By clicking on the *Browse strain by BacDive ID* the user can switch to the next *strain detail view* of a strain according to the BacDive identification numbering. The same functionality is offered by *Browse through all strains* on the BacDive entrance page (Figure 1c).

### Download selection and export

After queries have been submitted, search results are listed on a common results page (Figure 4). For CSV export all strain entries can be selected at once or particular strain entries can be selected individually. The list of selected strains is temporarily stored within the *download selection*. During an ongoing portal session these selected strains can be called anytime by clicking on the tab *DOWNLOAD SELECTION* in the BacDive navigation bar (Figure 1b).

## ACCESSIBILITY

The BacDive portal and its analysis functions are freely accessible at http://bacdive.dsmz.de. The content of BacDive is being continuously expanded and updated. The statistics of the data content of BacDive is published at the portal once a year as part of the annual BacDive major release in September. All features and functions of the BacDive portal are supported for the current versions of Firefox, Chrome and Internet Explorer. In addition to the web portal access, data of individual compilations of strains can be exported to CSV spreadsheet files. This export is limited to 200 strains for each export process. License information can be viewed at *IMPRINT/CONTACT* at the top right of the BacDive portal (Figure 1b). For all images presented in BacDive we disclose the copyright and licensing information directly at each *strain detail view*. The individual data annotation is currently tested at an initial alpha stage for BacDive. Invited annotators can obtain an account upon request. By directly logging on to the BacDive web portal these annotators can add information into the database. The new database content is stored together with the according reference information provided by the annotator.

## CONCLUSIONS

BacDive covers a broad range of information on a wide variety of bacterial and archaeal strains, in particular taxonomy, physiology, cultivation, geographic origin and INSDC sequence accession numbers for genomes and 16S rRNA. Additionally, the BacDive portal provides photomicrographs of the strains, especially for morphologically conspicuous bacteria such as the myxobacteria that form fruiting bodies. BacDive is an ideal central entry point for researchers looking for detailed information on distinct strains or in need of large-scale comparative analyses. The BacDive portal offers an easy-to-use *simple search* and a powerful *advanced search* functionality. By the *advanced search* maximal set of strains fitting in a well-defined, individual combined search pattern can be retrieved. The user may select particular strains and compile a *download selection* that can be exported in a CSV spreadsheet format. Hence BacDive derived data form a comprehensive basis for individual defined data analyses and is not limited to the current platform capabilities. The information in BacDive is predominantly annotated manually. Additionally, BacDive offers data retrieved by automatic text processing. These can be excluded in view and export functionalities. All information derived by automatic methods is consecutively manually reviewed.

With its particular features, BacDive will continue to become an increasingly comprehensive one stop information shop for cultured bacterial and archaeal metadata.

## FUNDING

Federal Ministry of Education and Research (BMBF) [01 LI 1001 C to J.O.]. Funding for open access charge: Leibniz-Institut DSMZ-Deutsche Sammlung von Mikroorganismen und Zellkulturen GmbH Inhoffenstraße 7B, 38124 Braunschweig, Germany.

*Conflict of interest statement*. None declared.
